# Surgical Uses of Picric Acid

**Published:** 1897-12

**Authors:** 


### SURGICAL USES OF PICRIC ACID.

C. M. Allen (*Brit. Med. Jour.*, Feb. 20, 1897), when experimenting with various therapeutic agents, has used picric acid (a concentrated solution in rectified spirits diluted with two parts of distilled water, and applied with a spray), and found it the most successful in allaying the irritation and stopping the discharge, and irritation from the discharge, from eczematous and ulcerated surfaces.

In the case of ulceration or eczema, or of ulceration and eczema of the leg, it is, subsequent to the application of the acid, covered with a medicated adhesive plaster.

In recent wounds, with or without great loss of substance, this treatment is equally beneficial, and is not so troublesome nor so unsuccessful as skin-grafting, where the size of the wound would render that advisable.

In cases of severe cellulitis arising from the introduction of some irritant poison, and when incision or amputation is necessary, the bleeding from the paralyzed and dilated radicles becomes almost uncontrolable. If the incision is packed with gauze wrung out of the picric solution the bleeding is soon controlled. For the oozing after the amputation the spray is much more successful than hot water, yet a combination of both may be used with great advantage, the water being used first (as it would decompose the acid if both were combined), and the spray applied immediately after.—*American Medico-Surgical Bulletin.*

